# Evolutionary Analysis of Plastid Genomes of Seven *Lonicera* L. Species: Implications for Sequence Divergence and Phylogenetic Relationships

**DOI:** 10.3390/ijms19124039

**Published:** 2018-12-14

**Authors:** Mi-Li Liu, Wei-Bing Fan, Ning Wang, Peng-Bin Dong, Ting-Ting Zhang, Ming Yue, Zhong-Hu Li

**Affiliations:** Key Laboratory of Resource Biology and Biotechnology in Western China, Ministry of Education, College of Life Sciences, Northwest University, Xi’an 710069, China; liumili@stumail.nwu.edu.cn (M.-L.L.); 201631689@stumail.nwu.edu.cn (W.-B.F.); wangning0810@stumail.nwu.edu.cn (N.W.); dongpb@stumail.nwu.edu.cn (P.-B.D.); zhangtt@stumail.nwu.edu.cn (T.-T.Z.); yueming@nwu.edu.cn (M.Y.)

**Keywords:** *Lonicera*, phylogenetic relationship, plastid genome, positive selection, repeat sequences

## Abstract

Plant plastomes play crucial roles in species evolution and phylogenetic reconstruction studies due to being maternally inherited and due to the moderate evolutionary rate of genomes. However, patterns of sequence divergence and molecular evolution of the plastid genomes in the horticulturally- and economically-important *Lonicera* L. species are poorly understood. In this study, we collected the complete plastomes of seven *Lonicera* species and determined the various repeat sequence variations and protein sequence evolution by comparative genomic analysis. A total of 498 repeats were identified in plastid genomes, which included tandem (130), dispersed (277), and palindromic (91) types of repeat variations. Simple sequence repeat (SSR) elements analysis indicated the enriched SSRs in seven genomes to be mononucleotides, followed by tetra-nucleotides, dinucleotides, tri-nucleotides, hex-nucleotides, and penta-nucleotides. We identified 18 divergence hotspot regions (*rps15*, *rps16*, *rps18*, *rpl23*, *psaJ*, *infA*, *ycf1*, *trnN-GUU-ndhF*, *rpoC2-rpoC1*, *rbcL-psaI*, *trnI-CAU-ycf2*, *psbZ-trnG-UCC*, *trnK-UUU-rps16*, *infA-rps8*, *rpl14-rpl16*, *trnV-GAC-rrn16*, *trnL-UAA* intron, and *rps12-clpP*) that could be used as the potential molecular genetic markers for the further study of population genetics and phylogenetic evolution of *Lonicera* species. We found that a large number of repeat sequences were distributed in the divergence hotspots of plastid genomes. Interestingly, 16 genes were determined under positive selection, which included four genes for the subunits of ribosome proteins (*rps7,* *rpl2*, *rpl16,* and *rpl22*), three genes for the subunits of photosystem proteins (*psaJ*, *psbC,* and *ycf4*), three NADH oxidoreductase genes (*ndhB*, *ndhH*, and *ndhK*), two subunits of ATP genes (*atpA* and *atpB*), and four other genes (*infA*, *rbcL*, *ycf1*, and *ycf2*). Phylogenetic analysis based on the whole plastome demonstrated that the seven *Lonicera* species form a highly-supported monophyletic clade. The availability of these plastid genomes provides important genetic information for further species identification and biological research on *Lonicera*.

## 1. Introduction

The genus *Lonicera*, which includes approximately 200 species, is a major component of the family Caprifoliaceae, comprising a large number of horticultural and economically important shrubs and tree species [1]. These plants are generally distributed in the temperate and subtropical regions of North America, Europe, Asia, and Africa [2], and about 100 *Lonicera* species are found in China. The majority of these species have important medicinal properties. For example, the extracts from *Lonicera* species have long been used for inflammation relief in some traditional Chinese medicines [3,4,5,6]. The research on *Lonicera* has mainly focused on the biological characteristics, classification, introduction, training, cultivation management, and chemical components of species [7,8,9,10,11]. The phylogenetic analysis based on the nuclear ribosomal internal transcribed spacer (ITS) and five chloroplast DNA regions demonstrated that *Lonicera* species diverged into two major lineages: *Chamaecerasus* and *Periclymenum* [12]. He et al. [13] first reported the whole plastid genome sequence of *Lonicera japonica*. However, the comparative characteristics of complete plastid genomes and phylogenetic evolution of *Lonicera* species are still poorly understood.

Generally, plant plastomes are closed cyclic DNA molecules, which typically have a quadripartite structure with a large single copy (LSC) region, a small single copy (SSC) region, and two identical copies of inverted repeats (IR) regions [14]. Some studies found that the plastid genomes of higher plants were highly conserved in genome organization, gene content, and order [14,15,16,17,18]. However, the repeat sequences in plastomes remained poorly understood for years. We now know that the repeat variations in plastid genomes often participate in diverse cellular functions including gene evolution, RNA editing, and gene mobility [19,20]. The repetitive sequences fall primarily into three classes: local repeats (tandem repeats and simple sequence repeats, SSRs), families of dispersed repeats (mostly transposable elements and retro-transposed cellular genes), and segmental duplications (duplicated genomic fragments). From an evolutionary point of view, the higher the level of organism evolution, the greater the proportion of repetitive sequences in the total plant genomes [21]. Long repeat sequences and SSRs were found scattered throughout the whole plastid genomes. Some studies found that most repeat sequences were located in the intergenic regions and intron regions, whereas few were located in the coding regions in angiosperm plastomes [22,23]. The diversity and versatility of functions of the repeated DNA sequences may be useful within the course of species adaptation to the environment.

Some studies showed that the repeat sequence variations and adaptive selection were the main evolutionary forces in the process of adaptive responses of species to the rapidly changed environments [24,25]. For instance, Yang et al. [26] found that positive selective action appears to have driven the functional divergence of the *CHS* gene in the process of speciation of *Dendranthema* (DC.) Des Moul. An evolutionary analysis of the plastid genes demonstrated that the chloroplast *rps4* gene might have, under positive selection, undergone diversification in the Cretaceous period after the rise of angiosperms [27]. The rapidly evolved plastid *matK* gene also experienced positive selection in some lineages of land plants [28]. However, the patterns of repetitive variations and adaptive evolution of the whole plastid genome sequence in most angiosperms remain largely unknown. 

In this study, we collected the whole chloroplast genomes of seven *Lonicera* species to detect the structure variations and performed a comparative analysis. The specific goals were: (1) to determine the distribution patterns of repeat sequence variations of plastid genomes, (2) to detect the positively selected genes in *Lonicera* plastid genomes, and (3) to identify the variant hotspot regions as potential molecular genetic markers for further population evolutionary studies.

## 2. Results

### 2.1. Characters of Plastid Genomes

**Figure 1:**
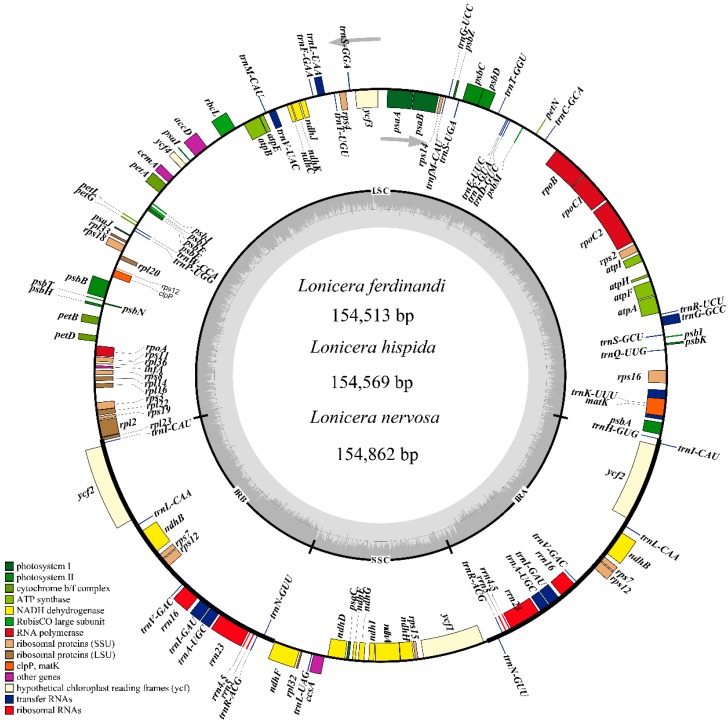
Chloroplast genome map for three *Lonicera* species. Genes located outside the outer rim are transcribed in a counterclockwise direction, whereas genes inside the outer rim are transcribed in a clockwise direction (as indicated by grey arrows). The colored bars indicate known different functional groups. The dashed gray area in the inner circle shows the percentage GC content of the corresponding genes. LSC, SSC, and IR denote large single copy, small single copy, and inverted repeat, respectively.

**Figure 2:**
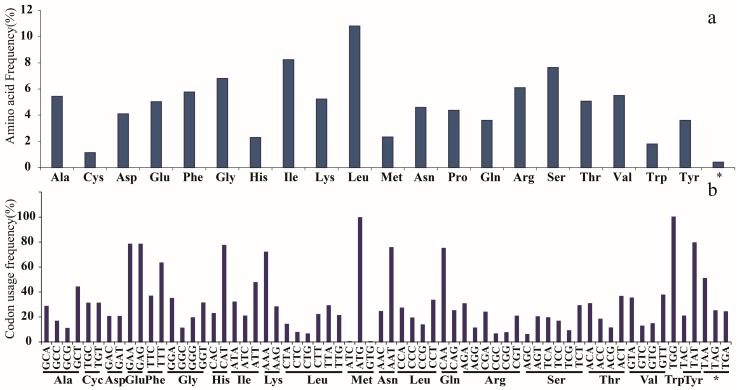
Amino acid (**a**) and codon usage (**b**) frequencies of the protein-coding sequences in the seven *Lonicera* chloroplast genomes. *: Termination codon.

The plastid genomes of seven *Lonicera* species ranged from 154,513 bp (*L. ferdinandi*) to 155,545 bp (*L. tragophylla*) in length (Table 1). The structure of the genomes was a typical quadripartite circular molecule composed of a LSC region of 88,505–89,288 bp, a SSC region of 18,552–18,766 bp, and a pair of IR regions of 23,646–23,791 bp (Figure 1, Table 1). The gene order and compositions were similar in the seven *Lonicera* species. The numbers and positions of genes in the seven *Lonicera* plastid genomes were also similar, which consist of 82 protein-coding genes, 37 tRNA genes, 8 rRNA genes, and 1 pseudogene (Table 2). Among the 128 genes, 20 genes contain introns comprised of 12 genes coding for proteins (*rps16*, *atpF*, *rpoC1*, *ycf3*, *rps18*, *rps12*, *rpl2*, *ycf*2 (two copies), *ndhB* (two copies), *ndhA*) and 8 tRNA genes (*trnK-UUU*, *trnG-GCC*, *trnL-UAA*, *trnV-UAC*, *trnI-GAU* (two copies), *trnA-UGC* (two copies)). The overall GC content is similar in the seven genomes, at about 38.4%. The overall GC content is unequally distributed across the plastid genome, which is the highest in the IR region (43.4%), followed by LSC (38.4%) and SSC (33.2%) regions. We summarized the codon usage and anticodon recognition patterns in the seven plastid genomes (Figure 2). Protein-coding genes comprise 25,110 amino acids in *L. hispida*, 25,178 amino acids in *L. nervosa*, and 25,222 amino acids in *L. ferdinandi*. Among these codons, those for leucine (10.8%) and isoleucine (8.2%) are the most common, and cysteine was the least frequently coded amino acid in the seven plastid genomes (Figure 2).

### 2.2. Repeat Sequences Analysis

**Figure 3:**
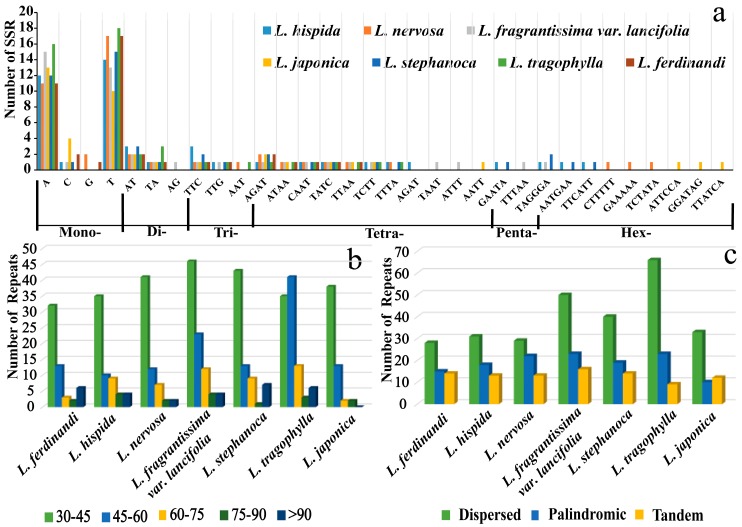
Analysis of repeated sequences in the seven *Lonicera* chloroplast genomes. (**a**) Frequency of selected motifs of simple sequence repeats (SSRs); (**b**) Frequency of repeat sequences of length >30 bp; (**c**) Composition of the repeats in seven *Lonicera* species.

We identified 313 SSR loci in the seven *Lonicera* plastids (Table S1, Figure 3a). Each species contains 41–49 SSRs (mean 45 SSRs). Among them, the mono-nucleotides repeat is the most common, which accounts for about 65.8% of total SSRs, followed by tetra-nucleotides (15.0%), dinucleotides (8.3%), tri-nucleotides (5.4%), and hex-nucleotides (4.5%). Penta-nucleotides (0.9%) were very rare across the plastid genomes. Mononucleotide SSRs are especially rich in A/T repeats (about 94%) in each *Lonicera* species. We found that the number of tetra-nucleotides SSRs is the largest in total SSRs, except mononucleotides. Most SSRs are located in noncoding sections (75%) and about 25% are in protein-coding regions (*ycf1*, *ycf2*, *atpB*, *rpoA*, *rpoB*, *rpoC1*, *rpoC2*, *ndhF*, *ccsA*, and *rpl23*) (Table S1). The numbers and distributions of all of the repeat types in the seven plastid genomes are similar and conserved (Figure 3, Tables S2 and S3). We identified 498 other types of repeats, which included tandem (91), dispersed (277), and palindromic (130) repeats in the seven *Lonicera* plastids. The number of dispersed repeats is more than that of palindromic repeats, and tandem is the lowest in these species. The length of the repeat units mainly ranges from 30 to 45 bp (Figure 3). These repeat sequences are mainly distributed in non-coding regions, whereas only a few are located in coding regions (*ycf2*, *ycf1*, *rpl20*, *rps18*, *ndhA*, *rps7*, and *ndhI*). A large number of repeat sequences are distributed around the pseudogene *accD* in these *Lonicera* species. 

### 2.3. Divergence Hotspots of Plastid Genomes

**Figure 4:**
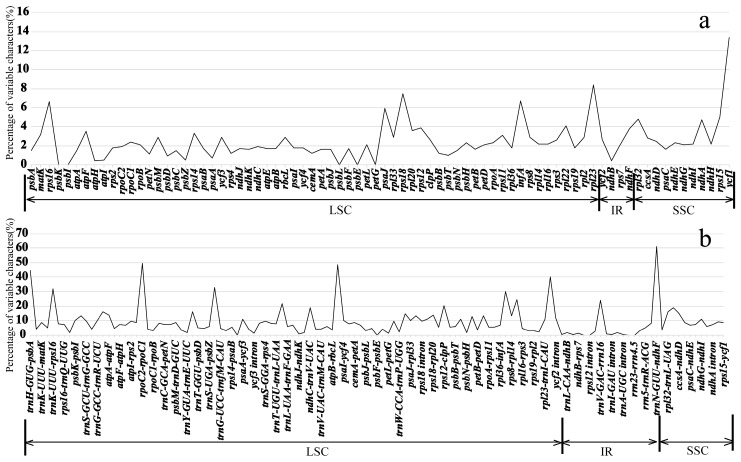
Percentages of variable characters in homologous regions among the chloroplast genomes of seven *Lonicera* species: (**a**) coding region and (**b**) noncoding region. The homologous regions are oriented according to their locations in the chloroplast genome.

**Figure 5:**
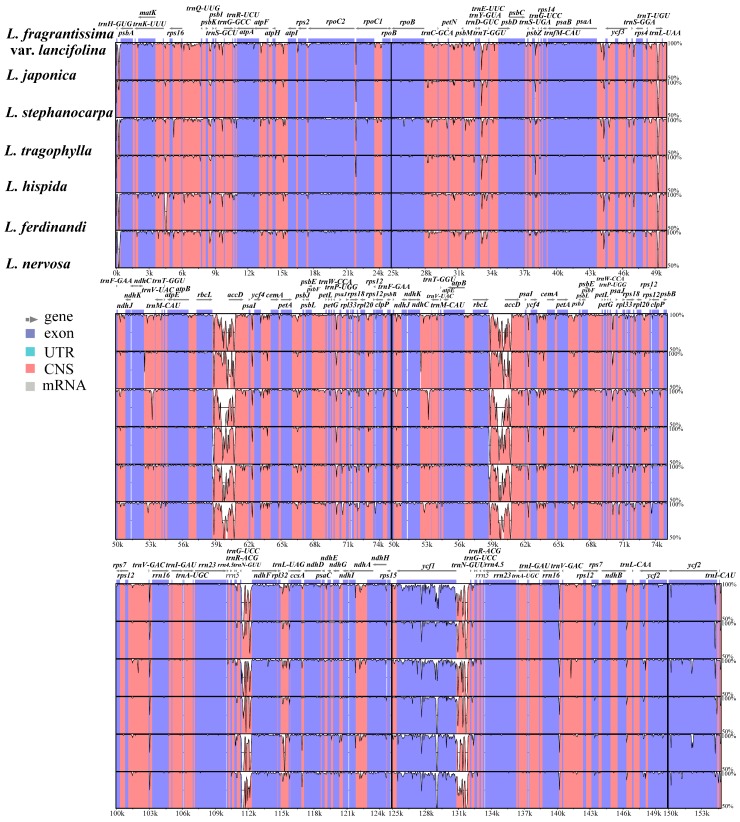
Sequence alignment of chloroplast genomes from seven *Lonicera* species. Sequences of chloroplast genomes were aligned and compared using the mVISTA program. The horizontal axis (x) indicates the coordinates within the chloroplast genome. The vertical scale (y axis) indicates the percentage identity, ranging from 50 to 100%. The grey arrows indicates the direction of each gene. Purple bars represent exons, orange bars show conserved non-coding sequences.

The coding genes, non-coding regions, and complete chloroplast genomes of seven *Lonicera* species were compared using the mVISTA program. To elucidate the level of sequence divergence, the percentages of variation were also calculated. As expected, non-coding regions and SC regions exhibited the higher levels of divergence than the coding and IR regions (Tables S4 and S5, Figure 4 and Figure 5). The percentage of variation in non-coding regions ranges from 0 to 61.3%, with an average of 9.37%, which is higher than that in the coding regions (ranging from 0 to 13.4%, an average of 2.50%). In coding regions, seven genes have the greatest variability (>5%): *rps15*, *rps16*, *rps18*, *rpl23*, *psaJ*, *infA*, and *ycf1*. Eleven intergenic regions have a percentage exceeding 20%: *trnN-GUU-ndhF*, *rpoC2-rpoC1*, *rbcL-psaI*, *trnI-CAU-ycf2*, *psbZ-trnG-UCC*, *trnK-UUU-rps16*, *infA-rps8*, *rpl14-rpl16*, *trnV-GAC-rrn16*, *trnL-UAA* intron, and *rps12-clpP* (Figure 5). 

**Figure 6:**
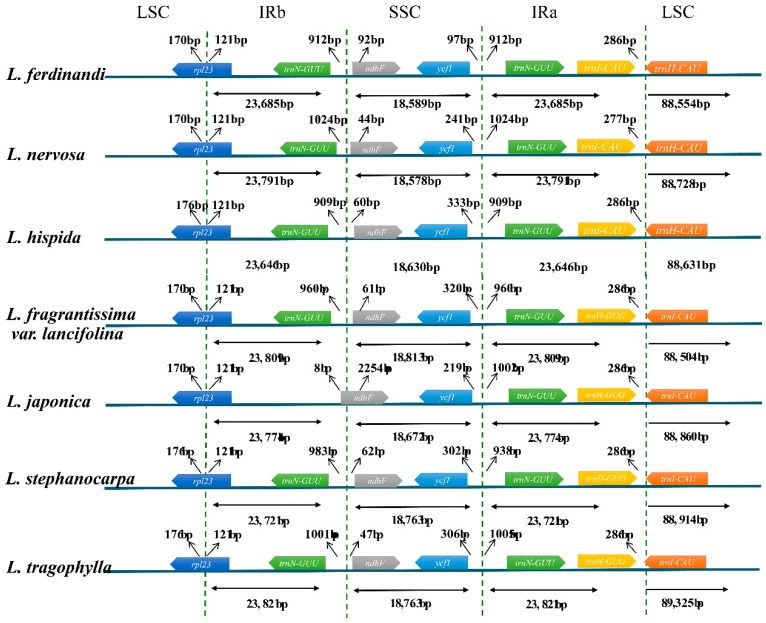
Comparison of the border positions of large single copy (LSC), small single copy (SSC), and inverted repeat (IR) regions in the chloroplast genomes in seven *Lonicera* species.

We analyzed the border structure of seven *Lonicera* plastid genomes. Detailed comparisons of the LSC, SSC, and IR regions are shown in Figure 6. The *rpl23* gene located in the IRb extended into the LSC region by about 170–176 bp. The *trnN* and *ndhF* genes are located in either side of LSC/IRb border and 969–1068 bp apart, whereas the *ndhF* gene is located in boundary of *L. japonica*. The *ycf1* gene is located in the SSC region, which ranges from 97 bp (*L. ferdinandi*) to 333 bp (*L. hispida*) away from the SSC/IRa border. IRa/LSC border performance is relatively stable, and the *trnH* gene is located 277–286 bp upstream of the IRa/LSC border.

### 2.4. Positive Selection Analysis

We detected 14 genes with positively selected sites via LRT tests (M0 vs. M3, M1 vs. M2, and M7 vs. M8) (*p* < 0.05, Tables S6 and S7), which included two genes for the subunit of ribosome protein (*rpl16, rpl22*), three subunits of the photosystem genes (*psaJ*, *psbC*, and *ycf4*), three NADH oxidoreductase genes (*ndhB*, *ndhH*, and *ndhK*), two subunits of ATP genes (*atpA* and *atpB,*) and four other genes (*infA*, *rbcL*, *ycf1*, and *ycf2*). Five genes (*ndhB*, *ndhK*, *rpl16*, *rpl22*, and *ycf4*) were detected in only one positively selected sites within Model 8, and the *ycf1* and *rbcL* genes were detected in more than two or, three selected sites for Model 8 than Model 2 with *p* > 95%, respectively. We detected the most selective sites (18) in the *ycf1* gene in the seven *Lonicera* plastid genomes.

### 2.5. Phylogenetic Analysis

**Figure 7:**
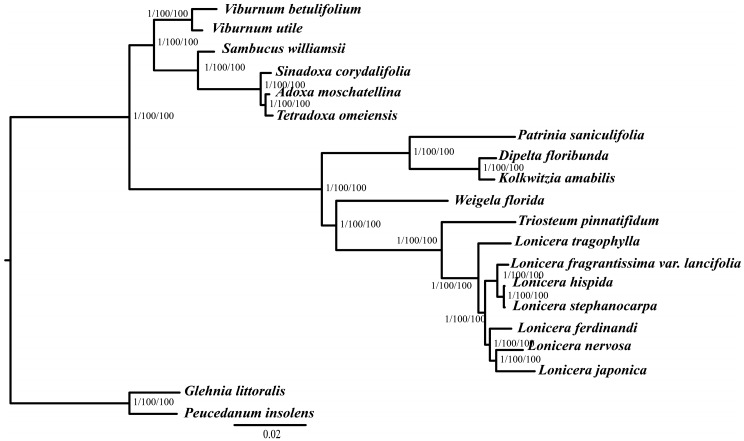
Phylogenetic tree obtained for seven *Lonicera* species based on the complete chloroplast genomes. The first number of the slashes on the branches shows the posterior probabilities according to Bayesian inference, the second number shows the bootstrap values obtained by maximum likelihood analyses, and the third number shows the bootstrap values obtained by maximum parsimony analyses.

To obtain an accurate phylogenetic relationship of *Lonicera* species, we performed multiple sequence alignments of 20 complete plastid genomes. The obtained topology is presented in Figure 7. The basic topologies were similar in the MP and ML analyses, which showed that the 18 Dipsacales species were divided into two parts, containing six Adoxaceae and 12 Caprifoliaceae species. Within Caprifoliaceae, *Patrinia saniculifolia* Hemsl. was placed as a sister clade to Linnaceae (*Dipelta floribunda* Maximowicz and *Kolkwitzia amabilis* Graebner) with 100% bootstrap values. We found that the seven *Lonicera* species formed a highly-supported monophyletic lineage. *L. tragophylla* separated first of seven *Lonicera* species. Three *Lonicera* species (*L. fragrantissima* var. *lancifolia*, *L. stephanocarpa*, and *L. hispida*) and the other three species (*L. ferdinandi*, *L. nervosa*, and *L. japonica*) formed a sister clade with high bootstrap value. 

## 3. Discussion

### 3.1. Features of Plastid Genomes

The available plastid genome sequences of most land plants have increased rapidly with the development of next generation sequencing (NGS) methods. However, the plastid genomes of *Lonicera* remained relatively limited, with only four species (*L. japonica*, *L. fragrantissima* var. *lancifolia*, *L. stephanocarpa,* and *L. tragophylla*) being reported [13,23]. Generally, most angiosperm plastid genomes are considered highly conserved in terms of their structure, gene content, and order [14]. In this study, we showed that the genome size of seven *Lonicera* species ranged from 154,513 to 155,545 bp, containing 82 protein-coding genes, 37 tRNA genes, 8 rRNA genes, and one pseudogene within quadripartite structure (LSC, 88,504–89,299 bp; SSC, 18,552–18,766 bp; and IR, 23,646–23,791 bp). The structure characteristics of the chloroplast genomes of these species are similar to those of most angiosperms [29]. In terms of GC content of the seven *Lonicera* plastids, the complete chloroplast genome had an overall GC content of ~38.4%, similar to the previously published *L. japonica* genome [13]. The GC content of IR regions is clearly higher than in the other regions, which are highly similar to most of land plants possibly due to the existence of the rRNA gene [30].

The pseudogenes in plastid genomes are functionless relatives of genes that have lost their ability to code and express a protein [31] relative to a complete gene. Although pseudogenes are not protein-coding DNA, these segment sequences may be similar to other kinds of noncoding regions, which may have a regulatory function [32] and have important roles in normal physiology and abnormal pathology [33]. In this study, we determined that the *accD* gene encoding a subunit of heteromeric acetyl-CoA carboxylase is a pseudogene in seven *Lonicera* species. The *accD* gene is known to be essential for leaf development in angiosperms [34]. Previous studies have shown that the *accD* gene has been lost in some angiosperm plastid genomes including Poales [35], Acoraceae [36,37], and Geraniaceae [38]. This gene may have played the main role in the physiological regulation in *Lonicera* species.

### 3.2. Repeat Sequence Variations

Previous studies suggested that repeat sequences may have played crucial roles in the rearrangement and stabilization of plastid genomes [39]. In the current study, we determined the dispersed, palindromic, and tandem repeats in seven *Lonicera* species, which showed that the number of tandem repeats is more than that of dispersed repeats, and palindromic repeats are the least common in these species. The majority of repeats were distributed in the intergenic spacer and intron regions, which is similar to those reported in other angiosperm lineages [26]. Variability in the copy number of SSRs in the chloroplast is generally polymorphic and can be used to analyze the population genetics and evolutionary studies at the inter- and intra-population levels [40]. We identified 313 SSR loci in the seven *Lonicera* plastid genomes. Most of these SSRs are located in noncoding regions (75%) and about of 25% are in protein-coding regions, similar to other angiosperms [41]. More tetra-nucleotide SSRs occur in the seven *Lonicera* plastomes. Among them, (AGAT)_3_ and (TATC)_3_ are shared by two *ycf2* genes. The (AGAT)_3_ repeat unit was also found in the pseudo-gene *accD* region in six *Lonicera* species, except for *L. tragophylla*. This large number of repeat sequences and SSRs is possibly related to the plastid genome size variation and divergence [42]. We identified 18 divergence hotspots (*rps15*, *rps16*, *rps18*, *rpl23*, *psaJ*, *infA*, *ycf1*, *trnN-GUU-ndhF*, *rpoC2-rpoC1*, *rbcL-psaI*, *trnI-CAU-ycf2*, *psbZ-trnG-UCC*, *trnK-UUU-rps16*, *infA-rps8*, *rpl14-rpl16*, *trnV-GAC-rrn16*, *trnL-UAA* intron, and *rps12-clpP*) in seven *Lonicera* plastid genomes. A large number of repeat sequences are also distributed in these divergence hotspot regions. These regions could be considered as potential molecular genetic markers for further study of population genetics and species evolution of *Lonicera*.

### 3.3. Positive Selection Analysis

Synonymous and nonsynonymous nucleotide substitutions are important markers for protein coding gene evolution. Generally, the rates of nonsynonymous and synonymous substitution in plant chloroplast genomes are relatively slow [43] due to the action of purifying and neutral selection [44]. In this study, we identified 14 protein-coding genes under positive selection. These genes included two small subunits of ribosome genes (*rpl16* and *rpl22*) that have been proven to be essential for the chloroplast ribosome development in plants [45]. Eleven genes (*ndhA*-*ndhK*) were found in the plastid genomes of most plants, encoding the NAD(P)H dehydrogenase (NDH) complex, which is involved in the I circulatory electron transport and chlororespiration, whereas three of these genes (*ndhB*, *ndhH*, and *ndhK*) were found to own selected sites. The family genes of *psa* and *psb*, and *ycf3* and *ycf4* genes were found to play vital roles in plant photosystem. The *psaJ* and *psbC* genes respectively belong to photosystem I and photosystem II. The *ycf4* gene forms modules that mediate PSI assembly as conserved chloroplast-encoded auxiliary factors [46]. The gene *infA* encodes translation initiation factor 1. It has been lost completely in some angiosperms [47,48] and is present as a pseudogene in the majority of angiosperms [47,48]. The *rbcL* gene was also found to play an important role as a photosynthetic electron transfer regulator, which is essential for photosynthesis [49]. We found *rbcL* gene possess nine sites under positive selection in these *Lonicera* species. A previous study also showed that the *rbcL* gene is often under positive selection in land plants [23,50]. The *ycf1* and *ycf2* genes are the largest plastid genes, encoding a protein that was part of the chloroplast inner envelope membrane protein translocon [51]. We identified 7 and 18 positively selected sites in the *ycf1* and *ycf2* genes, respectively. The current study also revealed that the positive selection of these two genes in angiosperm plants may be a common phenomenon [42].

### 3.4. Phylogenetic Relationship

In the previously phylogenetic results of Caprifoliaceae, Rehder [7] divided *Lonicera* species into two subgenera: *Lonicera* and *Caprifolium*. *Lonicera* subgenera contains four sections, *Coeloxylosteum*, *Isoxylosteum*, *Nintooa*, and *Isika*. In our study, phylogenetic analysis based on the complete plastid genomes showed that the seven *Lonicera* species form a highly-supported monophyletic lineage. *L. tragophylla* is separated from the seven *Lonicera* species. Some previously studies based on the partial nuclear and chloroplast DNA markers found that *L. ferdinandi*, *L. hispida*, *L. stephanocarpa*, and *L. fragrantissima* var. *lancifolia* belong to the *Isika* section, and *L. nervosa* belongs to *Rhodanthae* subsection, and *L. japonica* belongs to *Nintooa* [12]. These incongruent results may be due to the different sampling strategies and different molecular markers that were used. We also found that the three *Lonicera* species (*L. fragrantissima* var. *lancifolia*, *L. stephanocarpa*, and *L. hispida*) and the other three species (*L. ferdinandi*, *L. nervosa*, and *L. japonica*) form a sister clade with high bootstrap values. *L. ferdinandi* is closely related to *L. japonica* and *L. nervosa*. These findings are similar to previous morphological analyses of Caprifoliaceae species [7,12]. In conclusion, the results of phylogenetic analysis based on the plastid genomes greatly enhance our understanding of the evolutionary relationships among *Lonicera* species [52,53]. In the future, the more plastid genome datasets are needed to test the phylogenetic relationship and species evolution of *Lonicera* species.

## 4. Method

### 4.1. Sampling and Sequencing

Fresh leaves of three *Lonicera* species, *Lonicera nervosa* Maximowicz, *Lonicera ferdinandi* Franchet, and *Lonicera hispida* Pallas ex Schultes, were collected from Chunxin and Huating counties in Gansu province, China, in 2017. The dried plant samples and voucher specimens were deposited in the Key Laboratory of Resource Biology and Biotechnology in Western China (Shaanxi, China). The total genomic DNA was extracted from about 5 g of leaf tissue using a DNeasy Plant Mini Kit (Qiagen, Germany) according to the manufacturer’s instructions. About 5 ug purified DNA was used to construct paired-end libraries with 350 bp insert size and to sequence on an Illumina HiSeq 2500 platform by Novogene (Beijing, China). We downloaded the other four published *Lonicera* plastid genome sequences (*Lonicera japonica* Thunberg in Murray, *Lonicera fragrantissima* var. *lancifolia* (Rehder) Q. E. Yang Landrein, Borosova & J. Osborne, *Lonicera stephanocarpa* Franchet, and *Lonicera tragophylla* Hemsley) to employ the comparison analysis. 

### 4.2. Chloroplast Genome Assembly and Annotation

We trimmed the raw reads by removing the shorter and low-quality reads using NGSQCToolkit v2.3.3 software [54]. After clean reads of *L. nervosa*, *L. ferdinandi*, and *L. hispida* were assembled using MIRA 4.0.2 [55] with the complete plastid genome of closely-related species *L. japonica* (NC_026839) as the reference. To further assemble the whole plastid genomes, some ambiguous regions were extended using the MITObim v1.7 program [56] with a baiting and iteration method. The complete chloroplast genome sequences were imported into the online program Dual Organellar Genome Annotator (Dogma) [57] for annotation. The positions of starts, stops, introns, and exons were manually adjusted by comparison with homologous genes in other chloroplast genomes (*L. japonica*, *L. fragrantissima* var. *lancifolia*, *L. stephanocarpa*, and *L. tragophylla*). All tRNA genes were further confirmed using online tool tRNAscan-SE [58]. Eventually, the circular plastid genome maps were drawn using the bio-software OGDRAW [59]. The plastid genome sequences of the three *Lonicera* species and their raw reads were submitted to NCBI (accession numbers: MK176510-MK176512, SRR8269399, and SRR8269400).

### 4.3. Repeat Sequence Analysis 

In general, the long repeat contains dispersed, palindromic, and tandem repeats. In our study, the online software REPuter [60] was used to identify the dispersed and palindromic repeats with following conditions: (1) hamming distance of 1, (2) 90% or greater sequence identity, (3) and a minimum repeat size of 30 bp. The tandem repeats (>10 bp) were determined using the program Tandem Repeats Finder [61] with 2, 7, and 7 set for the alignment parameters match, mismatch, and indel, respectively. SSR loci were further detected using MISA software [62] with following thresholds: 10, 5, 4, 3, 3, and 3 repeat units for mono-nucleotide, di-nucleotide, tri-nucleotide, tetra-nucleotide, penta-nucleotide, and hexa-nucleotide SSRs, respectively.

### 4.4. Sequence Divergence Analysis

The complete plastid genomes of seven *Lonicera* species were compared using web-based program mVISTA [63] with *L. japonica* as the reference. To further identify the percentage of variable characters for each coding and non-coding region, the SNP sites were counted and positioned in the plastid genomes using DnaSP v5.0 [64].

### 4.5. Gene Selection Sites Analysis

The non-synonymous/synonymous substitution rate ratio (ω = dN/dS) is sensitive to the selection pressure in the evolution of protein level, and is particularly useful for identifying positive selection. A total of 75 protein-coding genes in *Lonicera* plastid genomes were extracted and compared using Genious R v9.0.5 [65] and MAFFT v7.0.0 [66]. The maximum likelihood phylogenetic tree was constructed using the program RAxML v7.2.8 [67] based on complete plastid genomes. The value of dN, dS, and ω for each gene exon were calculated using the site-specific model in the codeml program of Paml 4.7 [68]. In order to choose a more reliable model, we carried out the three likelihood ratio tests (LRT). The candidate sites of positive selection with significant support from posterior probability (p of (ω > 1) ≥ 0.99; Bayes Empirical Bayes approach) identified by M2 and M8 were considered further.

### 4.6. Phylogenetic Analysis

Phylogenetic analyses were performed on aligned data from 20 complete plastid genomes, which included 18 Dipsacales and two Apiaceae species, as demonstrated using Maximum parsimony (MP), Maximum likelihood (ML), and Bayesian inference (BI) analyses. Firstly, plastid genomes were aligned using MAFFT v7.0.0 [66] and the best-fitting model was selected using the MrModeltest 2.3 [69] through the Akaike information criterion (AIC). The ML and MP analyses were conducted using PAUP4 [70] with 1000 bootstrap replicates. BI analyses were performed using the program MrBayes v3.1.2 [71] with the settings as following: 1,000,000 generations Monte Carlo simulations (MCMC) algorithm, starting from random trees, and sampling 1 of every 1000 generations. Then 25% of all trees were burned using the software Tracer v1.6 [72].

## 5. Conclusions

In this study, we collected the complete chloroplast genomes of seven *Lonicera* species and determined the sequence variations and molecular evolution by comparative genomic analysis. The genus *Lonicera* plastomes exhibited a typical quadripartite DNA molecular structure, which is similar to those in other angiosperm species. A total of 498 repeats were identified in plastid genomes, which included tandem (130), dispersed (277), and palindromic (91) types of repeat variations. Simple sequence repeat (SSR) elements analysis indicated the enriched SSRs in seven plastomes to be mononucleotides, followed by tetra-nucleotides, dinucleotides, tri-nucleotides, hex-nucleotides, and penta-nucleotides. Interestingly, we determined eighteen divergence hotspot regions in these horticulturally- and economically-important *Lonicera* plastomes, which could be used as the potential molecular genetic markers for the further study of population genetics and phylogenetic evolution of *Lonicera* species. Selection pressure analysis showed that some plastid genes were under positive selection, which may played the important roles during the evolutionary process of *Lonicera*. Phylogenetic analysis based on the whole plastome revealed that the seven *Lonicera* species form a highly-supported monophyletic clade. The availability of these plastid genomes provides important genetic information for further species identification and evolutionary biological research on *Lonicera*.

## Figures and Tables

**Table 1:** The features of chloroplast genomes of seven *Lonicera* species.

Species	*L. ferdinandi*	*L. hispida*	*L. nervosa*	*L. fragrantissima* var. *lancifolia*	*L. stephanocarpa*	*L. tragophylla*	*L. japonica*
**No. of mapped reads**	180,643	159,034	158,808	–	–	–	–
**Average coverage**	149.1	419.2	198.4	–	–	–	–
**Total sequence length**	154,513	154,553	154,862	154,732	155,056	155,545	155,078
**Large single copy (LSC)**	88,554	88,631	88,728	88,504	88,912	89,299	88,858
**Inverted repeat (IR) region**	23,685	23,646	23,791	23,731	23,690	23,759	23,774
**Small single copy (SSC)**	18,589	18,630	18,552	18,766	18,763	18,728	18,672
**GC content (%)**	38.4	38.3	38.6	38.3	38.3	38.5	38.6
**GC content in LSC (%)**	36.9	36.8	36.9	36.8	36.8	37.0	37.1
**GC content in IR (%)**	43.4	43.4	43.5	43.3	43.4	43.5	43.5
**GC content in SSC (%)**	33.2	32.9	33.1	32.9	32.8	33.1	33.4
**No. of total genes**	128	128	128	128	128	128	128
**Protein-coding genes**	82 (4)	82 (4)	82 (4)	82 (4)	82 (4)	82 (4)	82 (4)
**tRNAs genes**	37 (7)	37 (7)	37 (7)	37 (7)	37 (7)	37 (7)	37 (7)
**rRNAs genes**	8 (4)	8 (4)	8 (4)	8 (4)	8 (4)	8 (4)	8 (4)
**Genes with introns**	20 (4)	20 (4)	20 (4)	20 (4)	20 (4)	20 (4)	20 (4)

Note: Numbers in brackets indicate genes duplicated in the IR regions.

**Table 2:** List of genes present in the chloroplast genomes of seven *Lonicera* species.

Gene Group	Gene Name				
Ribosomal RNA genes	*rrn16* (a)	*rrn23* (a)	*rrn4.5* (a)	*rrn5* (a)	*–*
Transfer RNA genes	*trnI-CAU* (a)	*trnI-GAU* (a)	*trnL-UAA*	*trnL-CAA* (a)	*trnL-UAG*
	*trnR-UCU*	*trnR-ACG* (a)	*trnA-UGC* (a)	*trnW-CCA*	*–*
	*trnV-UAC*	*trnV-GAC* (a)	*trnF-GAA*	*trnT-UGU*	*trnT-GGU*
	*trnP-UGG*	*trnfM-CAU*	*trnP-GGG*	*trnG-GCC*	*trnS-GGA*
	*trnS-UGA*	*trnS-GCU*	*trnD-GUC*	*trnC-GCA*	*trnN-GUU* (a)
	*trnE-UUC*	*trnY-GUA*	*trnQ-UUG*	*trnK-UUU*	*trnH-GUG*
Small subunit of ribosome	*rps2*	*rps3*	*rps4*	*rps7* (a)	*rps8*
	*rps11*	*rps12* (a)	*rps14*	*rps15*	*rps16*
	*rps18*	*rps19*	*–*	*–*	*–*
Large subunit of ribosome	*rp12*	*rp114*	*rp116*	*rp120*	*rp122*
	*rp123*	*rp132*	*rp133*	*rp136*	*–*
DNA-dependent RNA polymerase	*rpoA*	*rpoB*	*rpoC1*	*rpoC2*	*–*
Translational initiation factor	*infA*	*–*	*–*	*–*	*–*
Subunits of photosystem I	*psaA*	*psaB*	*psaC*	*psaI*	*psaJ*
	*ycf3*	*ycf4*	*–*	*–*	*–*
Subunits of photosystem II	*psbB*	*psbC*	*psbD*	*psbE*	*psbF*
	*psbH*	*psbI*	*psbJ*	*psbL*	*psbM*
	*psbN*	*psbT*	*–*	*–*	*–*
NADH oxidoreductase	*ndhA*	*ndhB* (a)	*ndhC*,	*ndhD*,	*ndhE*,
	*ndhG*,	*ndhI*	*ndhJ*	*ndhK*	*ndhF*
Subunits of cytochrome	*petA*,	*petB*,	*petD*	*petG*	*petL*
	*petN*	*–*	*–*	*–*	*–*
Subunits of ATP synthase	*atpA*	*atpB*	*atpE*	*atpF*	*atpH*
	*atpI*	*–*	*–*	*–*	*–*
Large subunit of Rubisco	*rbcL*	*–*	*–*	*–*	*–*
Maturase	*matk*	*–*	*–*	*–*	*–*
Envelope membrane protein	*cemA*	*–*	*–*	*–*	*–*
Subunit of acetyl-CoA	*accD* (b)	*–*	*–*	*–*	*–*
C-type cytochrome synthesis gene	*ccsA*	*–*	*–*	*–*	*–*

Note: (a) two gene copies in seven *Lonicera* species; (b) pseudogene in the seven *Lonicera* chloroplast genomes.

## Supplementary Materials

Supplementary materials can be found at http://www.mdpi.com/1422-0067/19/12/4039/s1.
