# Supplementary material for: Evolutionary Analysis of Plastid Genomes of Seven Lonicera L. Species: Implications for Sequence Divergence and Phylogenetic Relationships
Source: Int J Mol Sci. 2018 Dec 14;19(12):4039. doi: 10.3390/ijms19124039 (PMC6321470; doi:10.3390/ijms19124039)
Supplement: Supplementary file 1 [file ijms-19-04039-s001.zip › Table S7.docx]

**Table S7 Likelihood ratio test (LRT) of the variable ω ratio under different models.**

| gene | comparisons | Δl | df | p |
| --- | --- | --- | --- | --- |
|  | M0 vs M3 | 17.773922 | 4 | 0.00136617 |
| *atpA* | M1 vs M2 | 15.687789 | 2 | 0.0003921389 |
|  | M7 vs M8 | 16.442571 | 2 | 0.0002688692 |
|  | M0 vs M3 | 18.759976 | 4 | 0.0008760317 |
| *atpB* | M1 vs M2 | 14.129534 | 2 | 0.000854694 |
|  | M7 vs M8 | 16.042384 | 2 | 0.0003284283 |
|  | M0 vs M3 | 24.891375 | 4 | 5.290413e-05 |
| *infA* | M1 vs M2 | 15.341925 | 2 | 0.0004661689 |
|  | M7 vs M8 | 15.354562 | 2 | 0.0004632327 |
|  | M0 vs M3 | -0.000637 | 4 | 1 |
| *ndhB* | M1 vs M2 | 24.173005 | 2 | 5.635061e-06 |
|  | M7 vs M8 | 20.967302 | 2 | 2.799034e-05 |
|  | M0 vs M3 | 20.432368 | 4 | 0.0004102162 |
| *ndhH* | M1 vs M2 | 16.01611 | 2 | 0.0003327713 |
|  | M7 vs M8 | 16.177309 | 2 | 0.0003070026 |
|  | M0 vs M3 | 16.539114 | 4 | 0.002374849 |
| *ndhK* | M1 vs M2 | 14.352853 | 2 | 0.0007643945 |
|  | M7 vs M8 | 14.505722 | 2 | 0.0007081455 |
|  | M0 vs M3 | -1959.897434 | 4 | 1 |
| *psaJ* | M1 vs M2 | 20.738884 | 2 | 3.137679e-05 |
|  | M7 vs M8 | 21.190158 | 2 | 2.503892e-05 |
|  | M0 vs M3 | 19.072739 | 4 | 0.0007604985 |
| *psbC* | M1 vs M2 | -2.063803 | 2 | 1 |
|  | M7 vs M8 | 16.783078 | 2 | 0.000226778 |
|  | M0 vs M3 | 55.871595 | 4 | 2.133402e-11 |
| *rbcL* | M1 vs M2 | 33.851276 | 2 | 4.459527e-08 |
|  | M7 vs M8 | 33.991708 | 2 | 4.157138e-08 |
|  | M0 vs M3 | 17.294907 | 4 | 0.001693836 |
| *rpl16* | M1 vs M2 | 5.940532 | 2 | 0.05128967 |
|  | M7 vs M8 | 14.828333 | 2 | 0.0006026545 |
|  | M0 vs M3 | 9.124379 | 4 | 0.05806468 |
| *rpl22* | M1 vs M2 | 6.789901 | 2 | 0.03354221 |
|  | M7 vs M8 | 6.975087 | 2 | 0.03057589 |
|  | M0 vs M3 | 38.511081 | 4 | 8.789595e-08 |
| *ycf1* | M1 vs M2 | 20.933544 | 2 | 2.84668e-05 |
|  | M7 vs M8 | 21.179726 | 2 | 2.516987e-05 |
|  | M0 vs M3 | 40.783605 | 4 | 2.979889e-08 |
| *ycf2* | M1 vs M2 | 35.466434 | 2 | 1.988665e-08 |
|  | M7 vs M8 | 35.467937 | 2 | 1.987171e-08 |
|  | M0 vs M3 | 19.370664 | 4 | 0.0006645104 |
| *ycf4* | M1 vs M2 | 17.479865 | 2 | 0.0001600647 |
|  | M7 vs M8 | 17.54487 | 2 | 0.0001549458 |
